# Efficacy of bortezomib and thalidomide in the recrudescent form of multicentric mixed-type Castleman's disease

**DOI:** 10.1038/bcj.2015.12

**Published:** 2015-03-20

**Authors:** Q Lin, B Fang, H Huang, F Yu, X Chai, Y Zhang, J Zhou, Q Xia, Y Li, Y Song

**Affiliations:** 1 Henan Key Lab of Experimental Haematology, Henan Institute of Haematology, Henan Cancer Hospital affiliated to Zhengzhou University, Zhengzhou, China

A 16-year-old male patient was admitted to our hospital in March 2012 with chief complaints of systemic lymphadenopathy for >4 years with significant enlargement in the bilateral supraclavicular region for 1 month before admission. The patient had enlarged systemic lymph nodes, including the bilateral cervical, mediastinal and retroperitoneal lymph nodes, with no fever and night sweat initially in January 2008. Abdominal computed tomographic (CT) scan and ultrasonography revealed bilateral cervical, retroperitoneal lymphadenopathy and splenomegaly. The results of bone marrow cytomorphologic examination and biopsy were normal at that time. Biopsy of a left supraclavicular lymph node that was done in March 2008 at the first affiliated hospital of Zhengzhou University was suggestive of mixed-type Castleman's Disease. Enlarged lymph nodes significantly shrunk after 3 months of therapy with prednisone and interferon but they did not disappear. The patient had received CHOP(cyclophosphamide, hydroxydaunorubicin, oncovin and prednisone)-based regimen chemotherapy from June 2008. Lymphadenectasis was unpalpable and the splenomegaly was alleviated after treatment with six courses. Following this, the treatment was stopped after 6 months of interferon as maintenance therapy. Throughout the following 3 years, the patient was asymptomatic. However, in February 2012, he was admitted because of bilateral supraclavicular lymphadenopathy, which aggravated rapidly.

Physical examination revealed enlarged lymph nodes in the cervical, supraclavicular and axillary regions, the biggest of which was 6.4 cm × 3.9 cm in size in the left supraclavicular region. Splenomegaly (6.0 cm below the left costal margin) with no hepatomegaly was also palpated. Laboratory tests on admission showed a normal blood count. Urine analysis did not reveal protein traces (normal). The serum interleukin(IL)-6 level was 32 pg/ml (normal<5 pg/ml). No monoclonal immunoglobulin was detected in the serum by immunofixation electrophoresis. The serum *β*2-microglobulin and C-reactive protein (CRP) levels were 4.3 mg/l and 8.2 mg/l, respectively. Erythrocyte sedimentation rate was 38 mm in the first hour; anti-nuclear antibodies and rheumatoid factor were negative. Screening for human immunodeficiency virus, human herpes virus-8 (HHV-8), Epstein-Barr virus, and for hepatitis B and C viruses revealed negative results. All of the serum tumor markers tested (CEA, AFP, CA724, CA19-9, PSA and NSE) were negative. The thyroid function tests of T3, T4 and thyroid-stimulating hormone were normal. CT scans of the patient's chest and abdomen showed widespread lymphadenopathy in the thorax, axilla and retroperitoneal regions, as well as splenomegaly that was 14.9 cm × 4.7 cm in size. Electrocardiography test was normal.

Bone marrow biopsy was considered normal. Biopsy of a left supraclavicular lymph node revealed that the majority of germinal centers exhibited a tight/concentric pattern of follicular dendritic cell network, accompanied by vascular proliferation in the interfollicular space, and the typical onion-skin-like performance also appeared around germinal centers. (Figures 1a and b). An immunohistochemical study showed that CD20, CD79a and Bcl-2 were positive in the follicle, CD3 and CD5 were positive in the interfollicular space, CD10, CD38 and CD138 were scattered positive, CD34 was positive in the vein, and Vim was positive (Figures 1c and f), CK was negative, and Ki-67 was positive in about 10%. HHV-8 DNA was negative, as detected by nested PCR in the paraffin-embedded tissue specimens. The pathological diagnosis was suggestive of mixed hyaline-vascular and plasma cell type of Castleman's disease.

After obtaining signed informed consent from the patients, bortezomib combined Hyper-CVAD (fractionated cyclophosphamide, vincristine, doxorubicin and dexamethasone) routine chemotherapy was initiated. Bortezomib was given at the standard dose of 1.3 mg/m^2^ as an intravenous bolus on days 1, 4, 8 and 11, repeated at 21–28 days for a total of eight cycles; the chemotherapy consisted of the standard hyper-CVAD program that was given alternating hyper-CVAD with high-dose methotrexate and cytarabine for six cycles every 21–28 days. The patient did not present with a serious peripheral polyneuropathy and four grade neutropenia. Partial remission was reached after two cycles of treatment. The serum IL-6, *β*2-microglobulin and CRP level decreased to 11.3 pg/ml, 2.8 mg/l and 3.2 mg/l, respectively. A 75% decrease in lymph nodes and splenomegaly was noted. The treatment was well tolerated, and the patient was in complete remission after the fourth cycle when evaluated with the positron emission tomography-CT scan. Bortezomib was administered for another two cycles after six courses of chemotherapy. After treatment, the patient was in good condition; lymphadenectasis and splenomegaly were unpalpable, and serum IL-6, β2-microglobulin and CRP levels decreased to 3.1 pg/ml, 1.9 mg/l and 2.4 mg/l, respectively. Thalidomide was administered on sleep at a dosage of 200 mg/day as maintenance therapy for 6 months after the treatment with bortezomib. Compared with pre-therapeutic levels, lymph nodes and spleen remained stable and no significant changes were noted, and the patient was asymptomatic at 24 months' follow-up.

Multicentric Castleman's disease (MCD) is a subtype of Castleman's disease. It has been reported as a rare benign disease characterized by lymphocyte proliferation originally. Common therapies including steroid monotherapy and combined low-dose chemotherapy have been widely used, but there continue to be a number of patients in whom treatment is ineffective or in whom relapse occurs rapidly after the termination of treatment; in particular, the plasma cell subtype always has a highly aggressive feature.^1^ Progressive research on the pathogenesis of Castleman's disease has identified some novel agents including anti-IL-6 monoclonal antibody, humanized anti-human IL-6 receptor monoclonal antibody and rituximab for the MCD;^2, 3, 4^ a small number of MCD patients who were administrated bortezomib were also reported.^1, 5^

The patient in our study presented with progressing systemic lymphadenectasis at first, followed by significant bilateral supraclavicular lymph node enlargement. Immunohistochemistry and pathology of the left supraclavicular lymph node revealed the diagnosis of mixed-type Castleman's Disease in March 2008. After the common chemotherapy, lymphadenectasis was unpalpable and splenomegaly was alleviated. His condition was stable with no recurrence until February 2012. However, in February 2012, 3 years after the end of treatment, this patient relapsed. The same histopathology result was confirmed by the biopsy of the left supraclavicular lymph node. However, a more rapid progression of enlarged lymph nodes compared with 3 years ago was observed. To our knowledge, there is still no consensus treatment guideline for the relapsed MCD; obviously, it was difficult to achieve the desired effect for this relapsed patient through conventional chemotherapy. It was considered that the patient had a mixed hyaline-vascular and plasma cell type of MCD. Bortezomib has been reported to inhibit myeloma cell growth by NF-κB blockade and decrease IL-6 level. On the basis of the efficacy of bortezomib against B-cell malignancies and the experience in MCD reported in recent years,^1, 6^ we decided to treat this patient with bortezomib associated with Hyper-CVAD chemotherapy. Clinical improvement was achieved after completion of the second cycle of treatment. The patient was well tolerant to the treatment and showed a definitive clinical improvement and sustained reduction of IL-6, *β*2-microglobulin and CRP levels.

As is well known, thalidomide also has similar immunomodulatory and anti-angiogenetic properties to INF-alpha. in addition, thalidomide may decrease the serum IL-6 levels. Some studies reported that bortezomib and thalidomide were effective in the treatment of Castleman's Disease.^7, 8, 9, 10^ For this patient, in order to consolidate the effect, thalidomide was applied for 6 months as maintenance therapy. No treatment-related side effects occurred and the patient showed persistent clinical improvement.

Of note, in our study, administration of bortezomib-combined conventional chemotherapy followed by thalidomide successfully improved the condition of the patient with MCD, and induced significant and durable responses. Therefore, it is suggested that bortezomib and thalidomide could be used as salvage therapy for refractory MCD.

## Figures and Tables

**Figure 1:**
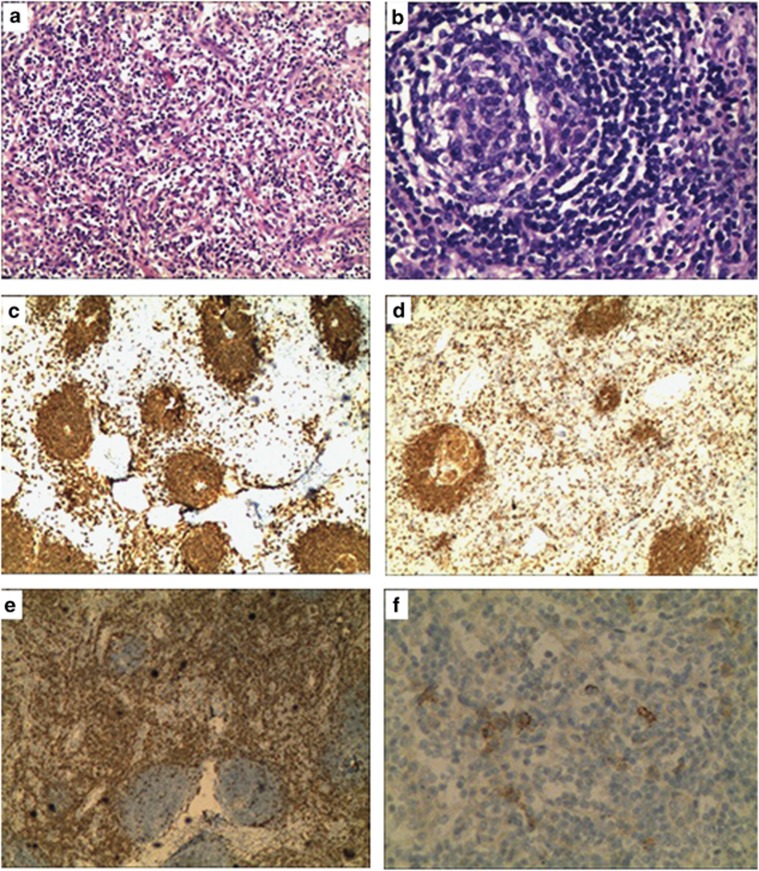
Hematoxylin and eosin (H and E) staining and immunohistochemical staining assessment of Castleman's disease. H and E staining: (**a**) It indicated proliferation of post-capillary venules in the interfollicular space and infiltration with plasma cells and eosinophils ( × 100). (**b**) It indicated follicles and a concentrically arranged mantle zone producing a characteristic 'onion peel' appearance ( × 200). Immunohistochemical staining for CD20 (**c**) and CD79a (**d**) was positive in the follicle, CD3 (**e**) was positive in the interfollicular space ( × 40) and CD138 was scattered positive (**f**) ( × 100).
